# MicroRNAs in cancer metastasis and angiogenesis

**DOI:** 10.18632/oncotarget.23115

**Published:** 2017-12-11

**Authors:** Weiyang Lou, Jingxing Liu, Yanjia Gao, Guansheng Zhong, Danni Chen, Jiaying Shen, Chang Bao, Liang Xu, Jie Pan, Junchi Cheng, Bisha Ding, Weimin Fan

**Affiliations:** ^1^ Program of Innovative Cancer Therapeutics, Division of Hepatobiliary and Pancreatic Surgery, Department of Surgery, First Affiliated Hospital, College of Medicine, Zhejiang University, Key Laboratory of Combined Multi-Organ Transplantation, Ministry of Public Health, Key Laboratory of Organ Transplantation, Zhejiang Province, Hangzhou 310003, China; ^2^ Department of Intensive Care Unit, Changxing People’s Hospital of Zhejiang, Zhejiang Province, Huzhou 313100, China; ^3^ Department of Anesthesiology, International Hospital of Zhejiang University, Shulan (Hangzhou) Hospital, Zhejiang Province, Hangzhou 310003, China; ^4^ Clinical Research Center, First Affiliated Hospital, College of Medicine, Zhejiang University, Zhejiang Province, Hangzhou 310003, China; ^5^ Department of Chemotherapy, Zhejiang Cancer Hospital, Zhejiang Province, Hangzhou 310003, China; ^6^ Department of Pathology and Laboratory Medicine, Medical University of South Carolina, Charleston, SC 29425, USA

**Keywords:** angiogenesis, cancer, microRNAs, metastasis, therapy

## Abstract

Cancer metastasis is a malignant process by which tumor cells migrate from their primary site of origin to other organs. It is the main cause of poor prognosis in cancer patients. Angiogenesis is the process of generating new blood capillaries from pre-existing vasculature. It plays a vital role in primary tumor growth and distant metastasis. MicroRNAs are small non-coding RNAs involved in regulating normal physiological processes as well as cancer pathogenesis. They suppress gene expression by specifically binding to the 3′-untranslated region (3′-UTR) of their target genes. They can thus act as oncogenes or tumor suppressors depending on the function of their target genes. MicroRNAs have shown great promise for use in anti-metastatic cancer therapy. In this article, we review the roles of various miRNAs in cancer angiogenesis and metastasis and highlight their potential for use in future therapies against metastatic cancer.

## INTRODUCTION

MicroRNAs (miRNAs, miRs) are a class of small endogenous non-coding RNAs, 21-25 nucleotides in length, which are highly conserved in evolution and usually exist as single copy or multi copy genes or as a gene cluster [1]. They are transcribed as long primary transcripts, which are subsequently processed by Drosha and Dicer [2]. Eventually, the mature miRNAs form a RNA-inducing silencing complex (RISC)-miRNA functional unit, which regulates the expression of nearly 30% of the known human genes [3]. The miRNAs base-pair with specific binding sites in the 3′-untranslated region (3′UTR) of their target messenger RNA (mRNA) and suppress gene expression at the post-transcriptional and translational levels [4]. The miRNAs are involved in a variety of biological processes such as cell proliferation, differentiation, apoptosis, survival, invasion, and migration [5–7]. Many studies have demonstrated that mutations in miRNA-encoding genes or deregulated expression of miRNAs are integral to many human diseases including cancers.

Angiogenesis is defined as the formation of new blood vessels from pre-existing capillaries or post-capillary venules [8]. Angiogenesis plays an important role in embryonic development as well as post-natal life [9]. Aberrant angiogenesis is central to many angiogenic diseases such as age-related macular degeneration (AMD) [10, 11], rheumatoid arthritis (RA) [12–14] and endometriosis (EM) [15, 16]. Aberrant angiogenesis is also critical for cancer metastasis [17–21].

Cancer is highly prevalent because of deterioration of the global ecological environment and the extension of life expectancy. In 2012, 14.1 million new cases of cancer were reported worldwide [22]. Unlike benign lesions, cancer subsequently metastasizes to distant tissues and organs, resulting in morbidity and mortality [23]. Although great advances have been made in the diagnosis and treatment of cancer metastasis, the prognosis of metastatic cancer patients remains extremely poor. Therefore, there is an urgent need to develop novel therapeutic approaches to treat cancer metastasis.

The role of miRNAs in anti-angiogenic therapy has emerged as a promising approach to treat metastatic cancers. In this review, we highlight recent findings about the role of miRNAs and their targets in cancer angiogenesis and metastasis. We also discuss the implications of miRNA-based therapeutic strategies targeting angiogenesis in metastatic cancer.

## BIOGENESIS OF MIRNA

**Figure 1:**
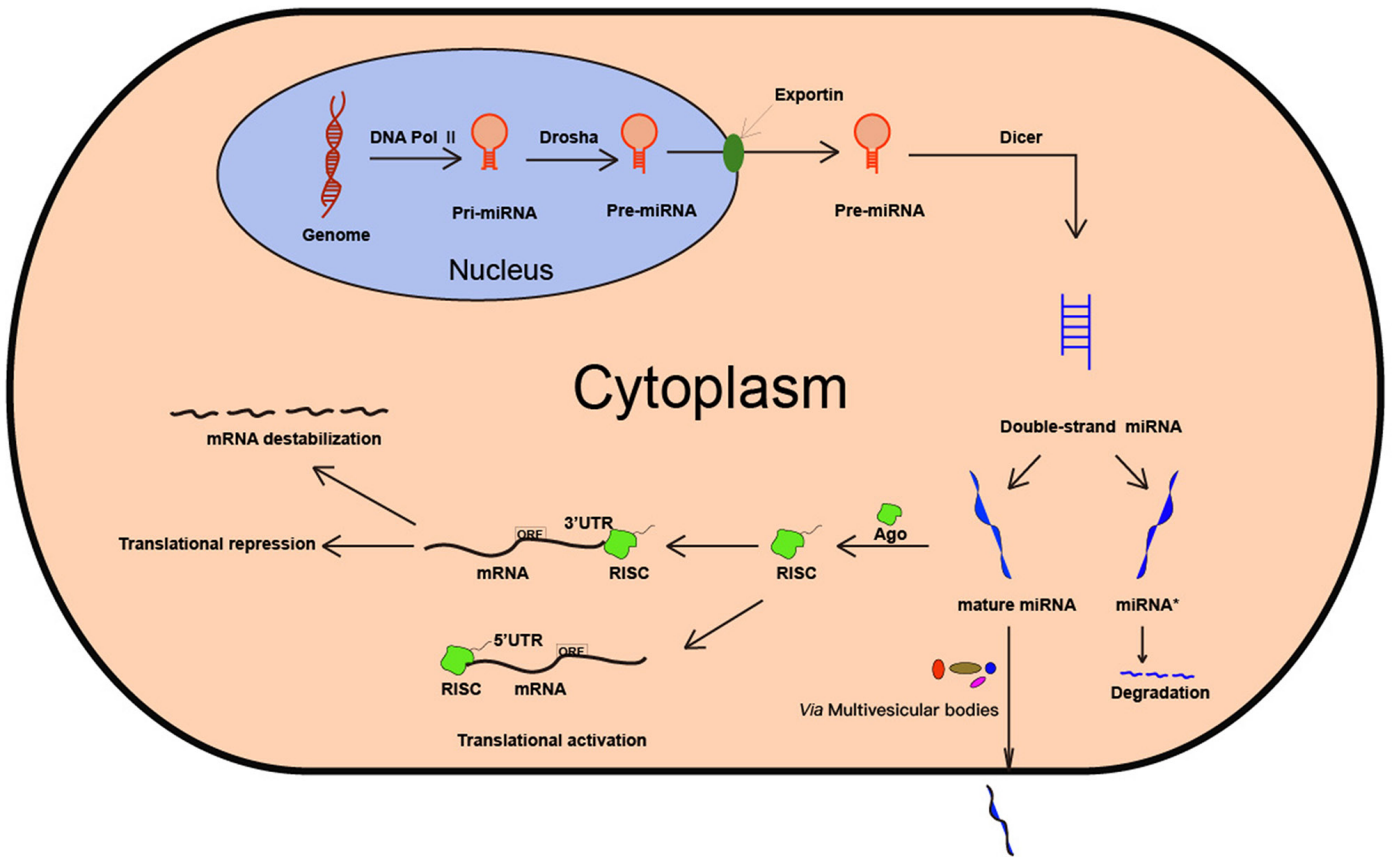
Biogenesis of miRNA. MiRNA is first transcribed by RNA Pol II. Then, the pri-miRNA is processed by the enzyme Drosha and Dicer. The mature miRNA is integrated into RISC, thereby leading to mRNA degradation, translational repression or translational activation.

MiRNAs are non-coding, small, single-stranded RNAs that are derived from the primary transcript called pri-miRNA, which is transcribed by RNA polymerase II [24]. The pri-miRNAs are characterized by the presence of a single or multiple imperfect hairpin structures with a stem of approximately 33 base-pairs [1]. Subsequently, the pri-miRNA precursor undergoes a two-step processing pathway, mediated by two ribonucleases, Drosha and Dicer belonging to the RNase III family [2]. In the nucleus, Drosha cleaves the pri-miRNA to generate an approximately 70 nucleotides long pre-miRNA, which is exported to the cytoplasm *via* an exportin-5-dependent mechanism [25–27]. In the cytoplasm, the pre-miRNA is further processed by Dicer to generate a mature, functional, double-stranded miRNA [28]. Then, the guide strand or mature miRNA is integrated into a multi-protein complex, RISC, which contains the argonaute (AGO) protein that plays a central role in RNA silencing. RISC uses the guide strand to target complementary 3′-UTR of mRNA *via* Watson-Crick base pairing [29, 30]. The other strand which is known as miRNA* or passenger strand is eventually degraded [31]. The miRNA binding to the 3′-UTR leads to mRNA degradation or translational repression, the extent of which is dependent on the degree of complementation. Besides, RISC can also target 5′-UTR of mRNA and activate translation [32]. The biogenesis of miRNA is shown in Figure 1.

## CANCER ANGIOGENESIS

**Figure 2:**
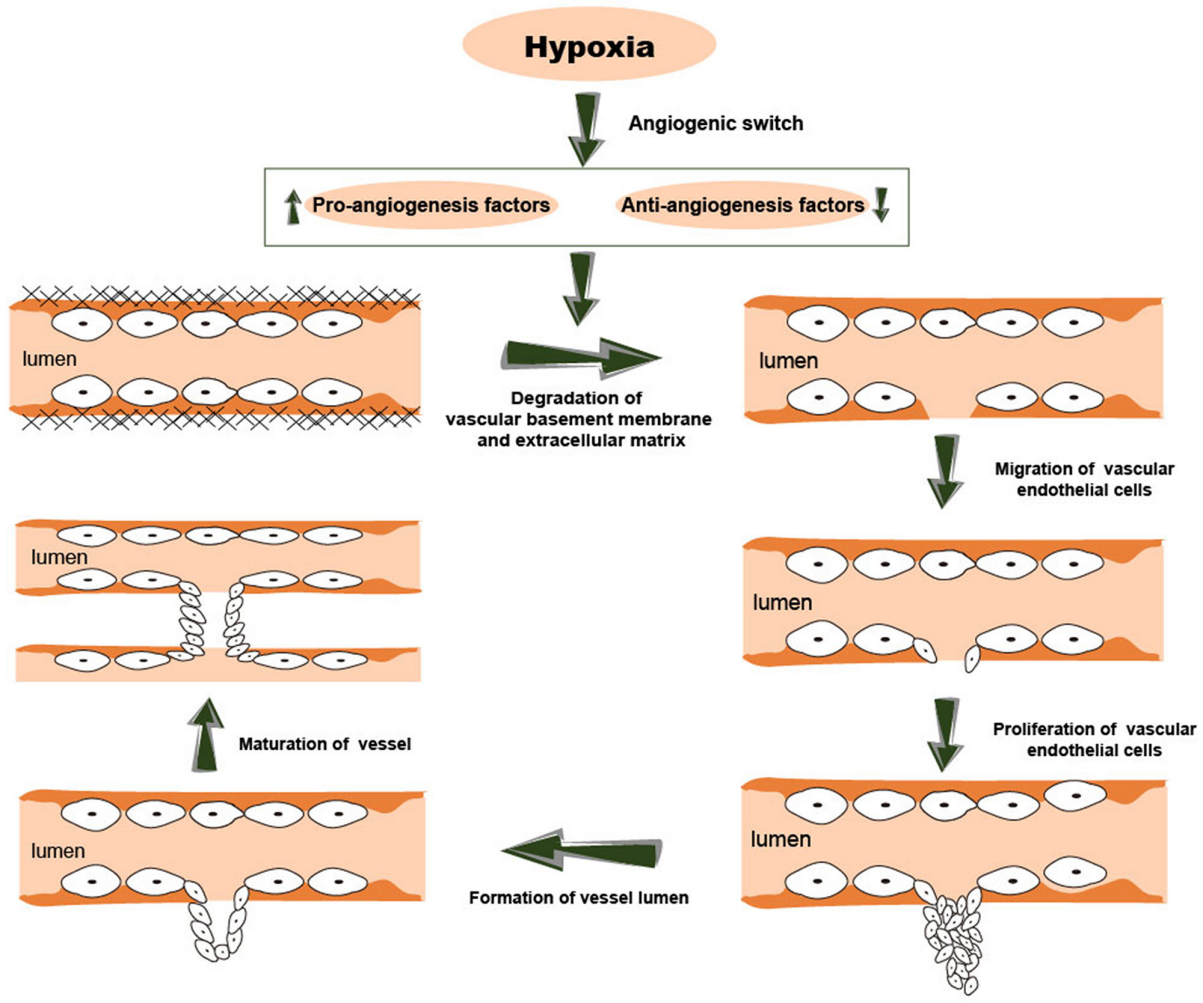
The process of cancer angiogenesis. This process is usually activated in a low oxygen microenvironment. Cancer angiogenesis involves multiple steps, including degradation of vascular basement membrane and extracellular matrix, proliferation and migration of vascular endothelial cells, formation of a new vessel lumen and vessel branches, and maturation of the new vessels.

Avascular, vascular and metastatic stages are the three stages of cancer. During the avascular stage, the tumor obtains nutrients and oxygen needed for growth by passive diffusion. However, the tumor growth is only about 1-2 mm in diameter without sufficient blood supply provided by angiogenesis [33]. Thus, angiogenesis is essential for uncontrolled growth of tumors. Initially, the pro- and anti-angiogenic factors are balanced in the tumor microenvironment. Cancer angiogenesis is similar to physiological angiogenesis and involves formation of new blood vessels through proliferation, migration and differentiation of endothelial cells (ECs) using pre-existing vascular structures [34]. Cancer angiogenesis is a complex multi-stage process involving degradation of vascular basement membrane and extracellular matrix, proliferation and migration of vascular endothelial cells, formation of a new vessel lumen and vessel branches, and maturation of the new vessel [35]. This process is activated due to low oxygen microenvironment in a growing cancer [35]. In response to the hypoxic environment, cancer cells undergo an angiogenic switch. Thus, the production of pro-angiogenic factors such as vascular endothelial growth factor (VEGF) and proteolytic enzymes [36] is increased and the induction of anti-angiogenic factors including angiopoietin 2 [8], angiostatin [37] and endostatin [38] is attenuated. Subsequently, the increased production of pro-angiogenic factors results in the activation of EC proliferation, differentiation, and migration. Eventually, a capillary network is successfully set up. The process of cancer angiogenesis is shown in Figure 2.

Unlike physiological angiogenesis, cancer angiogenesis is an inefficient process with sub-optimal perfusion, lack of vessel integrity and disorganized vessel network [39]. However, the newly-formed premature vessels provide the growing tumor tissue with adequate metabolites [40]. The immature structure of newly-formed blood vessels results in cancer cells gaining access to circulation. Moreover, the irregular and disorganized structure of blood vessels results in a high density vascular bed, which enhances the contact area between cancer and circulation, resulting in greater access for cancer cells to enter into circulation and promote distant metastases [40]. The number of metastasis sites positively correlate with the number of cancer cells initially entering the circulation [41]. Taken together, cancer angiogenesis not only acts as a bridge between primary cancer and circulation, but also plays a significant role in cancer metastasis. Metastatic cancer also undergoes three stages of development similar to primary cancer, which includes the avascular, vascular and metastatic stages. This vicious cycle results in morbidity and mortality of cancer patients. Therefore, cancer angiogenesis is pivotal to both initiation and progression of metastatic cancer.

## CANCER METASTASIS

Metastasis is closely related to poor prognosis of cancer patients [42]. It is the leading cause of cancer-related deaths and therefore critical for early diagnosis and treatment. About 50% of all cancer patients show clinically detectable metastasis at the time of diagnosis. However, micrometastases remain undetectable in a large number of cancer patients by the currently employed techniques [43].

**Figure 3:**
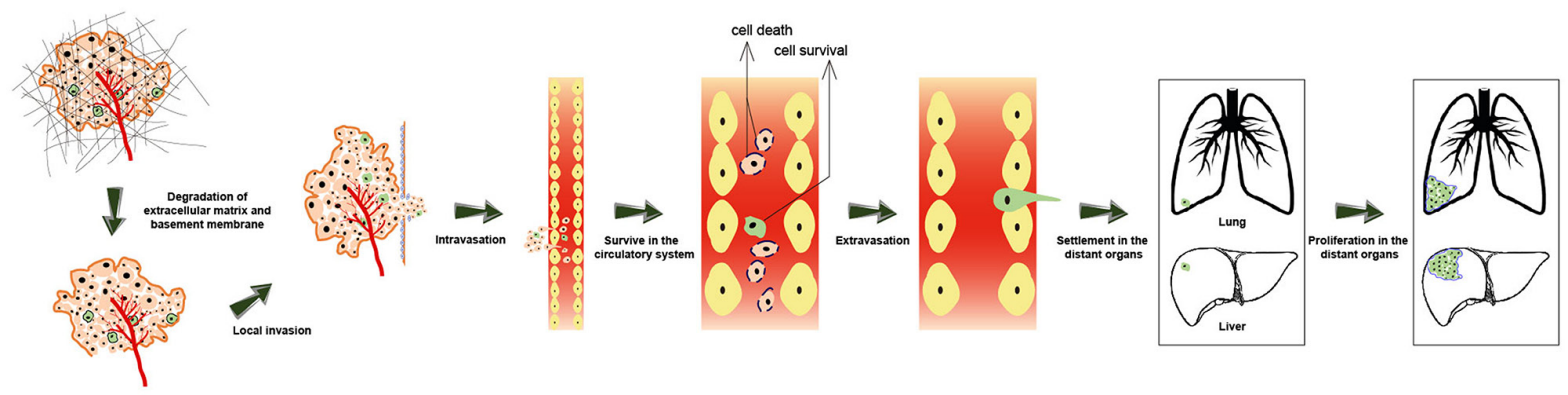
The process of cancer metastasis. A series of sequential steps are involved in cancer metastasis, such as alteration and rearrangement of cytoskeleton, degradation of extracellular matrix, local invasion, intravasation, transfer and survive in the circulatory system, extravasation, settlement and proliferation in a new organ (like lung and liver).

Metastasis is defined as the process by which cancer cells translocate from their primary cancer location to distant organs *via* the circulatory system or body cavities and subsequently establish a secondary cancer at the new tissue site [44]. As shown in Figure 3, cancer metastasis is an intricate process involving a number of sequential steps like (1) alteration and rearrangement of cytoskeleton, (2) degradation of extracellular matrix, (3) local invasion, (4) intravasation, (5) transport and survival in the circulatory system, (6) extravasation, and (7) settlement and proliferation in a new site [45]. Cancer metastasis has been well investigated in clinical studies. Despite being the central focus of clinical research, the specific mechanism of cancer metastasis has not yet been fully elucidated. It is widely thought that cancer metastasis occurs in association with the degradation of extracellular matrix [46], epithelial-mesenchymal transition (EMT) [47–49], overexpression of matrix metalloproteinases (MMPs) [50, 51], immune evasion [52], the homing of circulating cancer cells and cancer stem cells (CSCs) [53] as well as cancer microenvironment and angiogenesis [54].

## DYSREGULATED MIRNAS IN CANCER ANGIOGENESIS AND METASTASIS

### Anti-angiogenic and anti-metastatic miRNAs

#### MiRNAs targeting VEGF signaling

Vascular endothelial growth factor (VEGF) consists of VEGFA, VEGFB, VEGFC, VEGFD and placenta growth factor (PGF) [55]. Ectopic expression of VEGF partly accounts for cancer progression because of its involvement in cancer angiogenesis and metastasis [56, 57]. Many miRNAs regulate the VEGF expression. MiRNA-29c overexpression inhibits angiogenesis by downregulating VEGF [58]. Moreover, upregulation of miRNA-29c suppresses *in vitro* glioma cell migration and invasion due to reduced MMP-2 levels [58]. Wang *et al.* reported that the low expression of miRNA-195 promotes angiogenesis and metastasis of HCC *via* VEGF and the pro-metastatic factors, VAV2 and CDC42 [59]. Ghosh *et al.* showed that miRNA-199a-3p was downregulated in HCC tissues; its overexpression suppressed cancer growth, angiogenesis and lung metastasis by suppressing VEGFA, VEGFR1, VEGFR2, HGF and MMP2 [60]. Tu *et al.* showed that miRNA-497 inhibited breast cancer angiogenesis by targeting VEGFR2 [61]. Twist-induced downregulation of miRNA-497 promoted angiogenesis and metastasis of pancreatic cancer and was associated with high levels of VEGFA [62]. Besides, miRNA-497 suppressed HCC angiogenesis and metastasis by inhibiting VEGFA [63].

#### MiRNAs targeting HIF signaling

Hypoxia-inducible factor (HIF) is a transcriptional factor that responds to low oxygen levels. The dysregulation of HIF is vital for the formation of blood vessels in cancer, thereby accelerating cancer progression. Cha *et al.* showed that overexpression of miRNA-519c attenuated angiogenic activity of endothelial cells and suppressed angiogenesis and metastasis by reducing HIF-1α levels [64]. Therefore, cancer patients with high miRNA-519c levels had better prognosis [64]. Zhang *et al.* showed that miRNA-145 directly targeted HIF-2α, thereby inhibiting angiogenesis and metastasis of neuroblastoma [65]. Moreover, miRNA-145 negatively regulated gastric cancer angiogenesis and metastasis by suppressing Ets-1 transcription factor [66]. Mutations in p53 are positively correlated with cancer growth and angiogenesis because it regulates apoptosis, DNA repair, and cell-cycle progression *via* transcription of several miRNAs [67]. Yamakuchi *et al.* showed that p53 activated miRNA-107 transcription, which suppressed expression of HIF-1β, thereby inhibiting cancer angiogenesis, growth, and VEGF expression [68].

#### MiRNAs targeting angiopoietin-2 signaling

Angiopoietin-2 is a member of angiopoietins, which is mainly produced by ECs [69]. It facilitates VEGF-induced angiogenesis in multiple cancers. Therefore, inhibiting angiopoietin-2-related pathway suppresses cancer angiogenesis and metastasis [70, 71]. Ting *et al.* demonstrated that miRNA-542-3p inhibited angiopoietin-2 by directly binding to its 3′UTR [72]. Furthermore, miRNA-542-3p is a promising prognostic marker to monitor progression of breast cancer because its expression negatively correlates with clinical progression of stage III and stage IV breast cancer patients [72]. In HCC, downregulation of miRNA-542-3p is associated with intrahepatic metastasis and venous infiltration [73]. Fan *et al.* showed that reduced miRNA-543 levels correlated with colorectal cancer (CRC) metastasis [74]. In osteosarcoma, the expression of miRNA-543 was inhibited by connective tissue growth factor (CTGF), which resulted in increased Angiopoietin-2 levels that induced osteosarcoma angiogenesis [75].

#### MiRNAs targeting MMP signaling

Matrix metalloproteinases (MMPs) are calcium-dependent zinc-containing endopeptidases, which are essential for tissue remodeling associated with cancer angiogenesis and metastasis. MiRNA-9 induction inhibited MMP14 levels, which resulted in reduced angiogenesis, invasion and metastasis of neuroblastoma cells, both *in vitro* and *in vivo* [76]. Li *et al.* showed that MMP-14 was also a direct target of miRNA-181-5p in breast cancer cells, which resulted in attenuating breast cancer cell migration, invasion and angiogenesis [77]. Moreover, Ghosh *et al.* demonstrated that miRNA-199a-3p suppressed HCC growth, invasion, migration and angiogenesis by partially targeting MMP2 [60].

#### MiRNAs targeting LRP-6 signaling

Low-density lipoprotein receptor-related protein 6 (LRP6) and LRP5 are part of the LRP5/LRP6/Frizzled co-receptor, which is involved in the Wnt/β-catenin signaling [78]. LRP6 promotes cancer metastasis by participating in the canonical Wnt pathway in a variety of cancers such as triple negative breast cancer [79]. Fan *et al*. found that miRNA-454 inhibited cancer angiogenesis and metastasis by targeting LRP6 in pancreatic ductal adenocarcinoma (PDAC) [80]. The miRNA-454 overexpressing PDAC cells suppressed formation of capillary tube-like structures by HUVEC cells, thereby showing its role in inhibiting angiogenesis [80]. Xenograft experiments demonstrated decreased lung metastasis from miRNA-454 overexpressing PDAC cells than controls [80]. In another study, Du *et al*. showed that miRNA-126-3p partially suppressed angiogenesis and metastasis of HCC by targeting LRP6 [81]. Moreover, Sasahira *et al*. demonstrated that miRNA-126 inhibited metastasis in OSCC by suppressing VEGFA [82]. Therefore, miRNA-126-3p and miRNA-454 as well as LRP6 are potential targets for the treatment of cancer angiogenesis and metastasis.

#### MiRNAs targeting IL-6 signaling

Interleukin 6 (IL-6) is an inflammatory cytokine, which plays a role in cancer metastasis by downregulating E-cadherin [83]. Higher levels of serum IL-6 in patients with advanced or metastatic cancer suggest that it promotes metastasis. Yang *et al.* demonstrated that miRNA-26a inhibited *in vitro* HCC cell invasiveness and migration as well as *in vivo* metastasis by downregulating IL-6 [84]. Moreover, miRNA-26a also suppressed HCC angiogenesis [85]. MiRNA-451 is downregulated in human osteosarcomas and is implicated in suppressing angiogenesis and metastasis by targeting IL-6R [86]. Moreover, upregulation of miRNA-451 suppressed *in vitro* migration and angiogenesis of osteosarcoma cells [86]. Liu X *et al.* showed that miRNA-451 suppressed HCC angiogenesis by blocking the IL-6R/Stat3 pathway [87]. Thus, miRNA-451 demonstrates therapeutic potential as an anti-angiogenesis and anti-metastatic target.

### Other miRNAs

#### MiRNA-34a

CD44 antigen is a cell-surface glycoprotein that is relevant to cancer therapy and prognosis because of its role in cell-cell interactions as well as cell adhesion and migration [88]. Yu *et al.* reported low miRNA-34a levels in human bladder cancer tissues [89]. Moreover, overexpression of miRNA-34a inhibited angiogenesis and metastasis of bladder cancer cells by targeting CD44 [89]. Therefore, miRNA-34a and CD44 are potential anti-angiogenic and anti-metastatic therapeutic targets in bladder cancer patients.

#### MiRNA-101

Smits *et al.* showed that miRNA-101 inhibited proliferation, angiogenesis and migration of glioblastoma cells by targeting enhancer of zeste homolog 2 (EZH2)[90]. Moreover, Tang *et al.* showed that miRNA-101 was downregulated in nasopharyngeal carcinoma tissues and cell lines. They further showed that overexpression of miRNA-101 suppressed angiogenesis and lung metastasis by targeting Integrin subunit alpha 3 (ITGA3) [91].

#### MiRNA-124

Wang *et al.* demonstrated that upregulation of miRNA-124 attenuated *in vitro* migration, invasion and vasculogenic mimicry of bladder cancer cells by downregulating ubiquitin-like with PHD and RING finger domain 1 (UHRF1) [92]. In cervical cancer, miRNA-214 inhibited vasculogenic mimicry, migration and invasion by suppressing angiomotin-like protein, AmotL1 [93]. These studies imply that miRNA-124 and its related targets are potential targets for anti-angiogenic and anti-metastatic cancer therapy.

#### MiRNA-135a

MiRNA-135a is a tumor suppressor, which is reported to be downregulated in human prostate and gall bladder cancers [94, 95]. Cheng *et al.* reported that miRNA-135a levels were downregulated in gastric cancer tissues and cell lines [96]. They showed that miRNA-135a inhibited gastric cancer angiogenesis and metastasis by targeting the focal adhesion kinase (FAK), which regulates VEGF signaling [96]. Wang *et al.* showed low miRNA-135 expression in NSCLC tissues [97]. Overexpression of miRNA-135 suppressed *in vitro* NSCLC cell proliferation, invasion, migration and angiogenesis and induced cell apoptosis by blocking the IGF-1/PI3K/Akt signaling pathway [97].

#### MiRNA-218

Alajez *et al.* showed that miRNA-218 inhibited nasopharyngeal cancer progression by targeting survivin and SLIT2-ROBO1 pathway [98]. MiRNA-218 expression was silenced by DNA methylation in oral squamous cell carcinoma [99]. These findings suggested that miRNA-218 was a tumor suppressor. Zhang *et al.* showed decreased expression of miRNA-218 in gastric cancer [100]. MiRNA-218 inhibited gastric cancer angiogenesis and metastasis by downregulating ROBO1 [101]. These data suggested that miRNA-218 suppressed gastric cancer metastasis by inhibiting angiogenesis *via* a ROBO1-dependent mechanism.

#### MiRNA-320

Neuropilin 1 has been implicated in cancer angiogenesis and metastasis because of its interaction with VEGFA [102–104]. Neuropilin 1 is a target of miRNA-320 and its expression inversely correlates with miRNA-320 in oral squamous cell carcinoma (OSCC) [105, 106]. The overexpression of miRNA-320 suppresses OSCC angiogenesis [105]. Furthermore, inhibition of miRNA-320 accelerates the growth and metastasis of cholangiocarcinoma suggesting that it suppresses angiogenesis by depleting neuropilin 1 levels [106]. These results demonstrate the potential of miRNA-320 and neuropilin 1 as anti-angiogenic or anti-metastatic cancer therapeutic targets for OSCC.

#### MiRNA-409-3p

Angiogenin or ribonuclease 5 is a potent stimulator of angiogenesis [107, 108]. Weng *et al.* showed that overexpression of miRNA-409-3p decreased angiogenin mRNA and protein levels by binding to its 3′-UTR, thereby inhibiting fibrosarcoma vascularization and metastasis [109]. Conversely, knockdown of miRNA-409-3p increased fibrosarcoma progression [109]. Therefore, miRNA-409-3p is a potential target in fibrosarcoma therapy.

#### MiRNA-590-5p

Multiple studies have demonstrated the role of miRNA-590-5p in the initiation and progression of CRC [110–112]. Zhou *et al.* showed decreased miRNA-590-5p expression in human colorectal cancer (CRC) cells and tissues, demonstrating that miRNA-590-5p was a tumor suppressor in CRC [113]. Subsequent *in vivo* studies revealed that miRNA-590-5p knockdown promoted cancer angiogenesis, growth and lung metastasis, whereas its overexpression attenuated CRC progression by regulating nuclear factor 90 (NF90)/VEGFA signaling axis [113]. These data indicate that miRNA-590-5p is a potential target for human CRC therapy.

#### MiRNA-1301

There is increasing evidence that miRNA-1301 prevents angiogenesis and metastasis in hepatocellular carcinoma patients. MiRNA-1301 suppresses dissemination and metastasis of HCC cells *via* p53 [114]. Yang *et al.* demonstrated that miRNA-1301 was downregulated in HCC tissues and cell lines [115]. Moreover, miRNA-1301 targets B-cell CLL/lymphoma 9 (BCL9), which regulates β-catenin cofactors that are necessary for the transcription of Wnt target genes [115, 116]. Further studies demonstrated that miRNA-1301 inhibited hepatocellular carcinoma cell migration, invasion, and angiogenesis by decreasing Wnt/*β*-catenin signaling via BCL9 [115].

### Pro-angiogenic and pro-metastatic miRNAs

#### MiRNA-93

The miRNA-106b-25 cluster, which is a paralogue of miRNA-17-92 and miRNA-106a-363 clusters, consists of three mature miRNAs, namely miRNA-106b, miRNA-93, and miRNA-25 [117, 118]. The miRNA-106b-25 cluster is highly expressed in several human cancers and performs oncogenic function by suppressing P21 and Bim [119]. Jonathan *et al.* demonstrated that the miRNA-106b-25 cluster regulated the function of angiogenic bone marrow-derived stromal cells and endothelial cells and therefore was closely connected with angiogenesis [120]. Moreover, miRNA-93 promoted cancer growth and angiogenesis by targeting integrin-β8 [121]. Furthermore, miRNA-93 enhanced human breast cancer angiogenesis and promoted metastasis to lung tissue by suppressing the large tumor suppressor homology 2 (LATS2) protein, which is associated with cancer cell death [122]. Altogether, inhibition of miR-93 is a feasible approach to mitigate breast cancer angiogenesis and metastasis.

#### MiRNA-378

MiRNA-378 is widely recognized as an oncogene that promotes cancer growth, survival, angiogenesis and metastasis [123]. The levels of miRNA-378 are frequently increased in cancer tissue or serum of cancer patients and associated with poor prognosis [124, 125]. Lee *et al.* demonstrated that miRNA-378 enhanced U87 cancer cell survival and promoted cancer growth and angiogenesis [126]. SuFu, a negative regulator of Sonic Hedgehog (SHH) signaling, which facilitates large vessel formation by inducing the expression of pro-angiogenic cytokines including VEGF and Ang-1 is a miRNA-378 target [126]. FUS-1 is another direct target of miRNANA-378. FUS-1 overexpression reverses cancer cell survival and angiogenesis effects mediated by miRNA-378. Moreover, miRNA-378 is associated with brain metastasis of non-small cell lung cancer cells [127]. Furthermore, stable miRNA-378 overexpression increases non-small lung carcinoma growth, angiogenesis and metastasis by enhancing the expression of VEGF and Ang-1 [123]. These findings suggest that miRNA-378 is a potential target for anti-metastatic cancer therapy.

#### MiRNA-155

MiRNA-155 is frequently overexpressed in various types of human cancer and is linked to cancer angiogenesis and metastasis [128, 129]. Kong *et al.* found that the ectopic expression of miRNA-155 accelerated cancer angiogenesis and correlated with poor prognosis in triple-negative breast cancer [130]. MiRNA-155 overexpression induced network formation, proliferation, invasion and migration of human umbilical vein endothelial cells (HUVEC). There was an inverse correlation between miRNA-155 and Von Hippel–Lindau (VHL) expression. VHL overexpression rescued angiogenesis induced by miRNA-155, which indicated that miRNA-155 promoted angiogenesis by targeting VHL. The VHL protein is a component of the protein complex that possesses ubiquitin ligase E3 activity and is involved in the ubiquitination and degradation of HIF [131]. Petrovic *et al.* suggested that miRNA-155 promoted lymph node metastasis by investigating miRNA-155 levels in normal breast tissue, non-invasive and invasive breast carcinoma, and metastatic lymph nodes [132]. Johansson *et al.* showed that miRNA-155 targeted CCAAT-enhancer binding protein beta (C/EBPβ), which is a differentiation factor for the mammary epithelium and related to epithelial-mesenchymal transition (EMT) [133]. These results suggest that miRNA-155 is a potential therapeutic target to treat angiogenesis and metastasis of breast cancer.

#### MiRNA-494

MiRNA-494 is overexpressed in many cancers and plays a key role in cancer development and progression [134, 135]. Faversani *et al.* reported that miRNA-494 expression correlated with poor prognosis of lung cancer patients. Its overexpression enhanced motility and metastasis of lung cancer cells by activating NOTCH1 pathway and repressing PTEN/PI3K/AKT signaling [136]. Mao *et al.* showed that high miRNA-494 levels in lung cancer facilitated migration of vascular endothelial cells (ECs) and promoted angiogenesis by targeting PTEN, thereby activating Akt/e-NOS pathway. Moreover, intra-tumoral administration of miRNA-494 antagonists effectively suppressed lung cancer angiogenesis [137]. Therefore, miRNA-494 is a promising target for anti-angiogenic and anti-metastatic therapy for lung cancer patients.

#### MiRNA-296

The high expression of pro-angiogenic growth factor receptors on endothelial cells is a common feature of angiogenic blood vessels. These receptors include vascular endothelial growth factor receptor (VEGFR) and platelet-derived growth factor receptor (PDGFR), which are targets for anti-angiogenic therapies [138, 139]. Wurdinger *et al.* reported that miRNA-296 regulated levels of VEGFR2 and PDGFRß in angiogenic endothelial cells [140]. Moreover, they showed that Hepatocyte growth factor-regulated tyrosine kinase substrate (HGS), which degrades PDGFRß and VEGFR2 was highly repressed by miRNA-296 [140–142]. While miRNA-296 overexpression decreased HGS protein levels and increased PDGFRß and VEGFR2 levels that promoted angiogenesis, miRNA-296 antagonists attenuated cancer angiogenesis [140]. Additionally, clinical tissue microarrays showed that miRNA-296 was frequently upregulated in prostate cancer. Systemic delivery of miRNA-296 inhibitor decreased the incidence of pulmonary cancer metastasis by directly binding to the 3′UTR of intercellular adhesion molecule 1 (ICAM1) [143]. Therefore, miRNA-296 is a potential target in anti-angiogenic and anti-metastatic cancer therapy.

#### MiRNA-1246

MiRNA-1246 is a p53 transcriptional target, which participates in the regulation of the known anticancer functions of p53, such as activating DNA repair proteins and initiating apoptosis [144]. MiRNA-1246 promotes the development and progression of colorectal cancer [145]. Wang *et al.* demonstrated that miRNA-1246 overexpressing colorectal cancer cells exhibited higher invasive and migration capacity than controls [146]. In colorectal cancer tissues, miRNA-1246 levels were higher than adjacent normal tissues [147]. Yamada *et al.* demonstrated that promyelocytic leukemia protein (PML), a tumor suppressor protein required for the assembly of a number of nuclear structures [148] and a regulator of the Smad 2/3 signaling, was a direct target of miRNA-1246 [147]. Besides, CRC cell-derived microvesicles with miRNA-1246 facilitated CRC angiogenesis by downregulating PML. These findings show that miRNA-1246 is a potential therapeutic target to treat colorectal cancer angiogenesis and metastasis.

#### MiRNA-181a

MiRNA-181a is associated with T cell sensitivity, vascular development, cerebellar neurodegeneration and diabetes mellitus [149–152]. Sun *et al.* showed that miRNA-181a is oncogenic and upregulated in high grade chondrosarcoma by hypoxia [153]. The overexpression of miRNA-181a decreased regulator of G-protein signaling 16 (RGS16), which suppresses CXC chemokine receptor 4 (CXCR4) signaling. This resulted in increased expression of VEGF and MMP1 that promote chondrosarcoma angiogenesis and metastasis [153]. Thus, miRNA-181a is a potential therapeutic target for inhibiting chondrosarcoma angiogenesis and metastasis.

#### MiRNA-221 and miRNA-222

Epithelial-mesenchymal transition (EMT) in breast cancer is aberrantly activated by overexpression of miRNA-221 and miRNA-222, which target adiponectin receptor 1 (ADIPOR1) [154, 155]. Jikuzono *et al.* showed that miRNA-221/222 cluster was upregulated in metastatic minimally invasive follicular thyroid carcinoma (MI-FTC) [156]. Yang *et al.* reported that Tissue inhibitor of metalloproteinase 2 (TIMP2) was a direct target of miRNANA-221/222 in gliomas [157]. TIMP2 overexpression suppressed glioma angiogenesis and metastasis, which was enhanced by miRNA-221/222 [157]. Therefore, miRNA-221 and miRNA-222 are potential targets in the treatment of metastatic follicular thyroid carcinoma.

## CONCLUSIONS AND FUTURE PERSPECTIVES

Metastasis is the main cause of cancer-related deaths and is a big challenge in improving survival of cancer patients. Recent advances in the understanding of mechanisms underlying metastasis have opened up novel avenues to overcome the bottleneck in metastatic cancer therapy. Angiogenesis is a key step in cancer metastasis, which provides the channel for dissemination of cancer cells. Hence, blocking angiogenesis represents an effective therapeutic strategy for metastatic cancer. Anti-angiogenesis drugs have played a primary role in the treatment of a variety of metastatic cancers such as metastatic renal cell carcinoma [158]. However, the outcomes are unsatisfactory due to adverse effects such as bleeding and resistance to anti-angiogenic therapy [159, 160]. Therefore, novel alternatives of anti-angiogenic therapy are necessary. In the last decade, considerable evidence has accumulated about the involvement of miRNAs in cancer angiogenesis and metastasis. Table 1 shows few selected miRNAs that regulate cancer angiogenesis and metastasis. Studies have shown that dysregulation of these miRNAs greatly impacts cancer angiogenesis and metastasis. Huang *et al.* demonstrated that miRNA-30a negatively correlated with hematogenous metastasis of clear cell renal cell carcinoma by targeting angiogenesis-specific delta-like 4 (DLL4) [161]. This demonstrated that the regulation of miRNAs in angiogenesis contributed to cancer metastasis. Furthermore, rapid development of miRNA antagonists, mimics and delivery technologies has enabled the use of miRNAs in metastatic cancer therapy. However, the direct connection between the role of miRNAs in angiogenesis and cancer metastasis remains to be established. Besides, miRNA-based therapy is still not available in clinical settings. Nevertheless, with greater advances in technology, it is a matter of time before effective miRNA-based therapy is applied in the area of cancer angiogenesis and metastasis.

**Table 1:** Summary of dysregulated miRNAs in cancer angiogenesis and metastasis

Name	Expression	Tumor	Angiogenesis	Metastasis	Target gene	Reference
miR-29c	Down	Glioma	Suppression	Suppression	VEGF, MMP2	57
miR-195	Down	Hepatocellular carcinoma	Suppression	Suppression	VEGF, VAV2, CDC42	58
miR-199a-3p	Down	Hepatocellular carcinoma	Suppression	Suppression	VEGFA, VEGFR1, VEGFR2, HGF, MMP2	59
miR-497	Down	Hepatocellular carcinoma, breast cancer, pancreatic cancer	Suppression	Suppression	VEGFA, AEG-1, VEGFR2	60, 61, 62
miR-519c	Down	Lung adenocarcinoma, breast cancer	Suppression	Suppression	HIF-1alpha	63
miR-145	Down	Gastric cancer, neuroblastoma	Suppression	Suppression	HIF-2alpha, Ets1	64, 65
miR-107	Down	Colon cancer	Suppression	Suppression	HIF-1beta, VEGF, BDNF	67
miR-542-3p	Down	Breast cancer, hepatocellular carcinoma	Suppression	Suppression	ANG2	71, 72
miR-543	Down	Osteosarcoma, colorectal cancer	Suppression	Suppression	ANG2, KRAS, MTA1, HMGA2	73, 74
miR-9	Down	Neuroblastoma	Suppression	Suppression	MMP14	75
miR-181-5p	Down	Breast cancer, colon cancer	Suppression	Suppression	MMP14	76
miR-454	Down	Pancreatic cancer	Suppression	Suppression	LRP6	79
miR-126-3p	Down	Oral squamous cell carcinoma, hepatocellular carcinoma	Suppression	Suppression	VEGFA, LRP6, PIK3R2	80, 81
miR-26a	Down	Hepatocellular carcinoma	Suppression	Suppression	IL-6, HGF	83, 84
miR-451	Down	Osteosarcoma, hepatocellular carcinoma	Suppression	Suppression	IL-6R	85, 86
miR-34a	Down	Bladder cancer	Suppression	Suppression	CD44	88
miR-101	Down	Glioblastoma, nasopharygeal carcinoma	Suppression	Suppression	EZH2, ITGA3	89, 90
miR-124	Down	Bladder cancer, cervical cancer	Suppression	Suppression	UHRF1, AmotL1	91, 92
miR-135a	Down	Gastric cancer, non-small cell lung cancer	Suppression	Suppression	FAK, IGF-1	95, 96
miR-218	Down	Gastric cancer	Suppression	Suppression	ROBO1	99, 100
miR-320	Down	Oral squamous cell carcinoma, cholangiocarcinoma	Suppression	Suppression	Neuropilin 1	104, 105
miR-409-3p	Down	Fibrosarcoma	Suppression	Suppression	Angiogenin	108
miR-590-5p	Down	Colorectal cancer	Suppression	Suppression	NF90	112
miR-1301	Down	Hepatocellular carcinoma	Suppression	Suppression	BCL9	114
miR-93	Up	Breast cancer	Promotion	Promotion	Integrin-beta8, LATS2	120, 121
miR-378	Up	Non-small cell lung cancer	Promotion	Promotion	Sufu, Fus-1, HMOX1	125, 126
miR-155	Up	Breast cancer	Promotion	Promotion	VHL, C/EBPbeta	129, 131
miR-494	Up	Lung cancer	Promotion	Promotion	NOTCH1, PTEN	135, 136
miR-296	Up	Prostate cancer	Promotion	Promotion	HGS, ICAM1	139, 142
miR-1246	Up	Colorectal cancer	Promotion	Promotion	PML, CCNG2	145, 146
miR-181a	Up	Chondrosarcoma	Promotion	Promotion	RGS16	152
miR-221/222	Up	Thyroid carcinoma, glioma	Promotion	Promotion	ADIPOR1, TIMP2	153, 156

## Abbreviations

AdipoR1: Adiponectin receptor 1
AMD: Age-related macular degeneration
AmotL1: Angiomotin-like protein 1
Ang: Angiopoietin
BCL9: B-cell CLL/lymphoma 9
C/EBPbeta: CCAAT-enhancer binding protein beta
CTGF: Connective tissue growth factor
CXCR4: CXC chemokine receptor 4
EMs: Endometriosis
Ets1: V-ets erythroblastosis virus E26 oncogene homolog 1
EZH2: Enhancer of zeste homolog 2
FAK: Focal adhesion kinase
HGS: Hepatocyte growth factor-regulated tyrosine kinase substrate
HIF: Hypoxia-inducible factor
ICAM1: Intercellular adhesion molecule 1
IL-6: Interleukin-6
ITGA3: Integrin subunit alpha 3
LATS2: Large tumor suppressor homology 2
LRP6: Low-density lipoprotein receptor-related protein 6
MMP: Matrix metalloproteinase
NF90: Nuclear factor 90
PDGFR: Platelet-derived growth factor receptor
PGF: Placenta growth factor
PML: Promyelocytic leukemia protein
PTEN: Phosphatase and tensin homologue
RA: Rheumatoid arthritis
RGS16: Regulator of G-protein signaling 16
ROBO1: Roundabout homolog 1
SHH: Sonic hedgehog
SuFu: Suppressor of fused homolog
TIMP2: Tissue inhibitor of metalloproteinase2
UHRF1: Ubiquitin-like with PHD and RING finger domain 1
VEGF: Vascular endothelial growth factor
VHL: Von Hippel-Lindau
VPF: Vascular permeability factor
